# A60 DOWN WITH THE SHP-1: CHRONIC HELMINTH INFECTION DISRUPTS BILE ACID HOMEOSTASIS AND SIGNALLING RECEPTOR ACTIVATION IN THE MURINE SMALL INTESTINE TO POTENTIALLY IMPACT GUT IMMUNITY

**DOI:** 10.1093/jcag/gwac036.060

**Published:** 2023-03-07

**Authors:** J M Lane, T Brosschot, L Reynolds

**Affiliations:** Biochemistry & Microbiology, University of Victoria, Victoria, Canada

## Abstract

**Background:**

Helminths (parasitic worms) use immunomodulatory mechanisms to maintain their presence in the host intestines. Understanding how helminths reduce inflammation in the guts may lead to new therapeutic options for inflammatory bowel diseases. Our lab uses a helminth which infects the proximal small intestine of mice to identify new, potentially immunomodulatory molecules. In this project, we examined the impact of helminth infection on bile acids (BA)s; a class of metabolites that can function as immunomodulatory signalling molecules. To our knowledge, how helminth infection alters BA homeostasis, and the potential consequences for proximal small intestine immunity have not yet been reported.

**Purpose:**

Our aims were to determine whether helminth infection alters (1) the intestinal BA pool, (2) expression of BA transporters in the small intestine, and (3) expression and/or activation of the Farnesoid X receptor (FXR), a BA signalling receptor.

**Method:**

We used a murine model system to study the effects of the mouse-specific helminth *Heligmosomoides polygyrus* on BA homeostasis, BA transporter gene expression, and BA signalling receptors in the small intestine. Male and female littermate mice were used. Targeted metabolomics was used to assess the composition of the BA pool in the small intestine. Reverse transcription qPCR was used to quantify gene expression of BA transporters/signalling molecules in tissues. All data were segregated by sex of mice for data analysis.

**Result(s):**

We found that helminth infection disrupts the BA pool of mice, with consistent reductions in T-CDCA and T-αMCA during helminth infection. In the proximal small intestine, expression of the genes that encode the apical sodium dependant BA transporter (ASBT) and organic solute transporter alpha (OSTα) were unaffected during helminth infection, however expression of the organic solute transporter β (OSTβ) gene, which encodes half of the basal BA transporter, was decreased. Expression of *Shp-1*, an indicator of activation of BA receptor FXR, and expression of the *Fxr* gene were significantly decreased in both the proximal and distal small intestine. Lastly, expression of the gene encoding Gpbar1 trended upwards in the proximal and distal small intestine of female mice.

**Conclusion(s):**

Infection of mice with a proximal small intestine-dwelling helminth impacts BA homeostasis in the small intestine. Helminth infection decreases expression of a BA transporter in the proximal small intestine, although whether this contributes to or is caused by perturbations in the BA pool during infection remains to be seen. The downregulation of BA signalling we see during helminth infection in the proximal small intestine points to a potential functional impact on the host immune response which we now intend to explore. Understanding the immunomodulatory mechanisms used by helminths to dampen the host immune response could lead to further advancements in treating intestinal inflammatory disorders such as Crohn’s and Colitis.

**Disclosure of Interest:**

None Declared

